# Starch composition and functional properties of raw and pretreated anchote (*Coccinia abyssinica* (*Lam*.) *Cogn*.) tuber flours dried at different temperatures

**DOI:** 10.1002/fsn3.2687

**Published:** 2021-12-28

**Authors:** Adugna Mosissa Bikila, Yetenayet Bekele Tola, Tarekegn Berhanu Esho, Sirawdink Fikreyesus Forsido, Desta Fekadu Mijena

**Affiliations:** ^1^ Department of Post‐Harvest Management College of Agriculture and Veterinary Medicine Jimma University Jimma Ethiopia; ^2^ Department of Food Science and Nutrition Faculty of Agriculture Wollega University Shambu Ethiopia; ^3^ Department of Industrial Chemistry Addis Ababa Science and Technology University Addis Ababa Ethiopia; ^4^ Department of Plant Breeding College of Agriculture and Veterinary Medicine Jimma University Jimma Ethiopia

**Keywords:** anchote flour, drying temperature, functional properties, pretreatment, starch composition

## Abstract

Anchote (*Coccinia abyssinica* (*Lam*.) *Cogn*.) is an indigenous tuber crop produced in southwest part of Ethiopia. As an indigenous and underutilized tuber, limited scientific information is available about the characteristics of dried anchote powder. In this study, attempts were made to investigate the starch composition and functional properties of flours produced from the raw and pretreated tuber dried at different temperatures (60, 80, and 100°C). The results showed that both pretreatment and drying temperature significantly (*p <* .05) affected the amylose/amylopectin ratio, pH, total soluble solids (TSS), water absorption capacity (WAC), oil absorption capacity (OAC), water absorption index (WAI), water solubility index (WSI), swelling power (SP), total polyphenols content (TPC), and total flavonoid content (TFC). The treatment combinations result in varied ranges of pH (5.70–6.47), TSS (5.37–10.8 °Brix), WAC (2.42–4.21 g/g), OAC (0.94–1.44 g/g), WAI (3.40–5.42 g/g), WSI (11.40%–20.37%), SP (4.56–7.20 g/g), foaming capacity (FC) (3.31%–33.33%), foam stability (FS) (1.89%–20.00%), amylose content (AC) (14.18%–36.11% ), TPC (0.22–0.80 mg GAE/g), and TFC content (0.12–0.44 mg CE/g). The blanched and boiled anchote flours dried at relatively lower drying temperature exhibited better WAC, SP, and WAI than the raw. Considering the determined parameters, the flour from the tuber can be used as an ingredient in different food formulations.

## Graphical abstract

Starch composition and functional properties of raw and pretreated (blanched and boiled, raw as a control) anchote (*Coccinia abyssinica* (*Lam*.) *Cogn*.) tuber flours dried at different temperatures were evaluated. The results showed that both pretreatment and drying temperature significantly (*p* < .05) affected the amylose/amylopectin ratio, pH, TSS, WAC, OAC, WAI, WSI, SP, TPC, and TFC of the flours. The blanched and boiled anchote tuber flours dried at lower temperature exhibited better functional property relative to the raw.

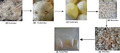

## 1 INTRODUCTION

Anchote (*Coccinia abyssinica* (*Lam*.) *Cogn*.) is a potentially productive and nutritious starchy tuberous crop indigenous to Ethiopia, but its utilization is restricted to the southwest part of the country. The most common edible part of anchote is its tuber, but its leaf and young fruit are also consumed in some areas (Fekadu, 2011). The total yield of the tuber was reported to be about 15–18 tons/ha, which is comparable with that of sweet potato and potato (Fekadu, 2014), and usually harvested after 3–5 months of planting (Abera & Haile, 2015; Mekbib & Deressa, 2016). Compared with other common root and tuber crops, the crop is highly nutritious and contains valuable nutrients such as carbohydrates, protein, fiber, and different minerals particularly calcium (Ayalew, 2016; Parmar et al., 2017).

However, it is an underutilized crop due to limited processing and value addition except for the traditional boiling of the tuber in excess water (Parmar et al., 2017). Under such conventional cooking practice, the tuber is hard to cook and takes a long time compared with other starchy tubers such as cassava and sweet potato (Parmar et al., 2017), resulting in increased demand for energy to cook. In addition, boiling for an extended time may affect the nutrient composition of the tuber due to thermal degradation and leaching out in boiling water. Furthermore, like other tuber crops, the tuber of anchote cannot be stored for an extended period due to its perishable nature and loss in quality. Underground storage was commonly practiced to avoid postharvest losses, but this prevents producers from using the same field for other crops.

These limitations hindered the extensive utilization of the tuber in different forms and different parts of the country and elsewhere. Converting the tuber into semiprocessed shelf‐stable and easy‐to‐use products such as flour is necessary for safe storage, loss reduction, broader utilization, convenience, and commercialization. However, the conversion of the tuber into flour may need predrying treatments and drying practice, which may affect the product's composition and other native properties. The effect of drying temperature and predrying treatments on the drying kinetics of the anchote tuber and on the nutritional composition and thermal properties of its flour was recently reported (Bikila et al., 2020, 2021). However, the starch composition and functional properties of the flour as affected by predrying treatment and drying temperature have not yet been studied well.

Understanding the starch composition and functional properties of anchote flour is crucial if one plans to use it for different purposes by further processing. Native starches have unique starch composition and functional properties, which are influenced by the starch's structural characteristics, shape, and botanical source. In addition to this, starchy products usually do not possess the desirable functionality after pretreatment or processing, which may limit their utilization in food and nonfood applications (Gunaratne et al., 2016). Alteration in starch composition could also affect functional properties of the dried flour due to complex interaction between the composition, structure, molecular conformation, and nature of food components.

Consequently, the characterization of the starch composition and functional properties of the anchote tuber flour is necessary for extensive utilization and commercialization of the product for different purposes. The flour made from the tuber can be used as an ingredient to formulate various products for different social groups. Therefore, this study aimed to investigate the effect of predrying treatments and drying temperature on the starch composition, and physicochemical and functional properties of anchote tuber flours.

## 2 MATERIALS AND METHODS

### 2.1 Materials

The anchote tuber used for this study was *Desta 01*, the first improved variety released by Debre zeit Agricultural Research Center under the Ethiopian Institute of Agriculture Research (DZARC/EIAR) in 2018.

### 2.2 Research design

A 3 × 3 factorial experiment in a completely randomized design was employed to run the experiment. The first factor included predrying treatments in three levels (untreated (raw), blanched, and boiled tubers), the second factor was drying temperature in three levels (60, 80, and 100°C), and the experiment was replicated three times.

### 2.3 Sample preparation

**FIGURE 1:**
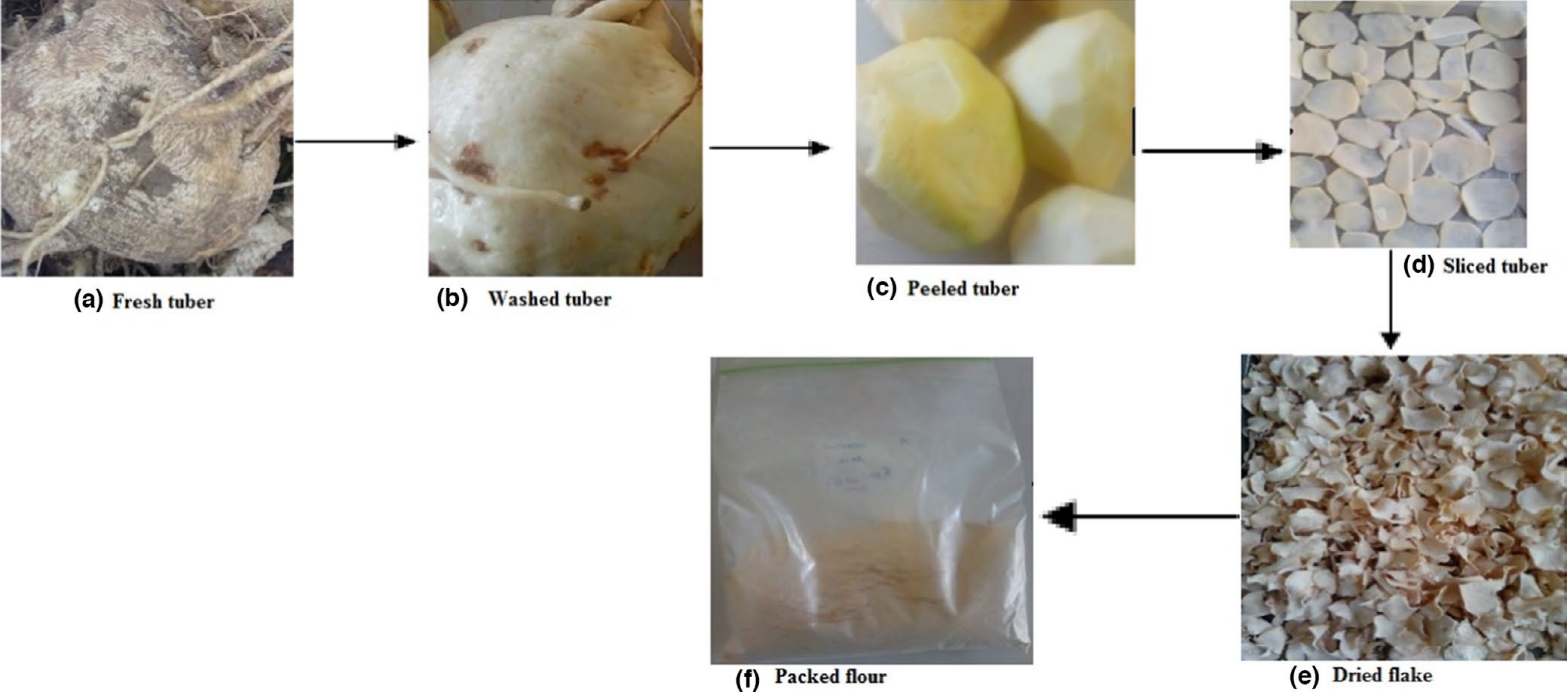
Anchote flour preparation procedure from its tuber, from one of the treatments to represent the process

Anchote flour samples were prepared as per the primary steps shown in Figure 1. Fresh anchote tuber was washed in running tap water to remove the adhered substances, then peeled off using stainless steel knives, washed further with tap water, and sliced into ~2 mm thickness. After mixing the slices, the samples were then grouped into three: raw tuber, tuber subjected to blanching in hot water (98 ± 2°C) for 5 min to avoid enzymatic browning, and that boiled in water for 30 min. After blotting the slices in tissue paper, the samples were then dried in a convective hot air‐drying oven (*Labquip, Leicester LE67 5FT, England*) at 60, 80, and 100°C until constant weight was obtained. Dried tuber slices were then ground into flour, homogenized, sieved through a 500‐*μ*m mesh, packed in a moisture‐proof polyethylene bag, and stored at −4°C until analysis.

### 2.4 Determination of starch composition

#### 2.4.1 Starch extraction

Starch extraction was carried out using the alkaline method described by Tattiyakul et al. (2006). One part of anchote flour (g) was dispersed in five parts of 0.05% (w/v) NaOH solution (mL) and let to stand for 2 hr. The suspension was screened through a 200‐mesh sieve, and the filtrate was centrifuged (*SIGMA 2‐16KC, India*) at 3000 *g* and 4°C for 10 min. The supernatant was then decanted, the sediment was resuspended in five parts of distilled water, and centrifugation was carried out. The washing step using distilled water was repeated four times. The sediment was finally dried in a hot air convective oven (*LABQUIP, LEICESTER LE67 5FT, England*)) at 40°C for 16 h, ground, and sieved through a 100‐mesh sieve, and the starch flour were preserved for further analysis.

#### 2.4.2 Amylose and amylopectin contents

Amylose and amylopectin of anchote starch were determined according to Arueya and Ojesanmi (2019). Accordingly, 0.10 g of the starch sample was weighed into a 100‐ml volumetric flask, and 1 ml of 99% ethanol and 9 ml of 1 M NaOH solution were added. The contents were mixed thoroughly, and the solution was heated for 10 min in boiling water to gelatinize the starch. After cooling, the solution was made up to the mark with distilled water and shaken thoroughly. Then, 5 ml of the starch solution in the 100‐ml volumetric flasks was treated with 1.0 ml of 1 M acetic acid and 2.0 ml iodine solution (0.2%). The final solution was diluted to the mark with distilled water, and the absorbance was read using a spectrophotometer (*V‐630; JASCO, Japan*) at 620 nm. The absorbance of the blank solution was deducted from each sample, and percent amylose content was determined using the following equation, and the difference estimated in the amylopectin content (%).

(1) AmyloseContent(%)=3.06×A×20%

where *A* is the absorbance read at 620 nm, 3.06 is the conversion factor calculated as milligram amylose in unit absorbance, and 20 is the dilution factor.

### 2.5 Determination of functional properties

#### 2.5.1 pH

The pH of the flour samples was determined using a pH meter (*pH‐016; HINOTEK, China*) calibrated against standards of pH 7 and 4. Five grams of each sample was dispersed in 25 ml of distilled water to prepare the sample. The solution was stirred constantly for 30 min, and pH measurement was taken with frequent shaking until stable reading.

#### 2.5.2 Total soluble solids

Total soluble solids (TSS) (*°Brix*) of the flour sample was estimated using 10% aqueous solution of each flour using a hand refractometer (*IP65; Eclipse Range, UK*) according to the method described in Thriveni et al. (2019). A clear solution was obtained by filtering the sample using multiple layers of muslin cloth. From the solution, 2–3 drops were placed on a calibrated refractometer, and the TSS was measured in *°Brix*.

#### 2.5.3 Water absorption capacity

According to the method described by Witono et al. (2014), Water absorption capacity (WAC) was determined gravimetrically with slight modification. One gram of the flour sample was immersed in 10 ml of distilled water at room temperature (21°C) and shaken to mix well using a sealed centrifuge tube, after which it was left to reach the maximal swelling. After 30 min, the mixture was centrifuged at 805 *g* for 30 min, at room temperature (21°C). Then, the swollen sample was separated by decantation and weighed. Finally, WAC was calculated as g H_2_O/g dry flour using the following equation:

(2) $$\text{WAC}\left(\frac{g}{g}\right)=\frac{m2‐m1}{m1}$$

where *m*_2_ is the sample's weight with the absorbed water and *m*_1_ is the initial weight of the dry flour sample.

#### 2.5.4 Oil absorption capacity

Oil absorption capacity (OAC) was determined according to the method described by Achy et al. (2017). One gram of the flour sample was immersed in 10 ml of soybean oil at room temperature (21°C), shaken to mix well, and then left to reach the maximal absorption. After 30 min, the mixture was centrifuged at 1811 *g* for 30 min. Then, the clear supernatant was decanted, and the sediment was weighed. Finally, OAC was calculated using the following equation:

(3) $$\text{OAC}\left(\frac{g}{g}\right)=\frac{m2‐m1}{m1}$$

where *m*_2_ = sample's weight with the absorbed oil, whereas *m*_1_ = initial weight of the dry flour.

#### 2.5.5 Water absorption index

The water absorption index (WAI) was determined according to the method described by Yousf et al. (2017). The anchote flour (2 g) was suspended in distilled water (20 ml) at room temperature (21°C) for 30 min and gently stirred and then centrifuged at 805 *g* for 15 min. The supernatant was poured into a tarred evaporating dish to be used for WSI (g/g) determination. The remaining sediment was weighed, and WAI was calculated as grams of the gel obtained per gram of solid flour.

(4) $$\text{WAI}=\frac{\text{Weight of sediment with absorbed water}}{\text{Weight of dry solid flour}}$$

#### 2.5.6 Water solubility index

The water solubility index (WSI) was determined according to the method reported by Yousf et al. (2017). The supernatant obtained from WAI analysis was dried in an oven at 100°C to constant weight, and the dry weight of the dissolved solid was taken. Then, WSI was expressed as a percentage of the original weight of the flour sample (2 g).

(5) $$\text{WSI}\;\left(\%\right)=\frac{\text{Weight of dissolved solid in supernatant}}{\text{Weight of dry solids}}\times 100$$

#### 2.5.7 Swelling power

The swelling power (SP) of the flour samples was determined according to the procedure reported by Kusumayanti et al. (2015). A 0.1 g sample was heated in 10 ml distilled water in a water bath (HH‐S4 Laboratory Thermostatic Digital Water Bath, China) at 60°C for 30 min with constant mixing. The samples were centrifuged at 229 *g* for 15 min at room temperature (21°C), the precipitated part was weighted, and SP was calculated using the following equation:

(6) $$\text{SP}\left(\frac{g}{g}\right)=\frac{\text{Weight of sedimental paste}\;(g)}{\text{Weight of the dry sample}\;(g)}$$

#### 2.5.8 Foaming capacity and foam stability

The foaming capacity (FC) and foam stability (FS) of anchote flours were evaluated according to the method reported by Chandra et al. (2015). One gram flour of the sample was added to 50 ml of distilled water at 30 ± 2°C in a 100‐ml graduated cylinder. The suspension was mixed and shaken for 5 min to make the foam. The volume of foam at 30 s after whipping was expressed as foam capacity using the following equation.

(7) $$\text{FC}\left(\%\right)=\frac{\text{Volume of foam AW}‐\text{Volume of foam BW}}{\text{Volume of foam BW}}\times 100$$

where FC is the foam capacity, AW is the after whipping, and BW is the before whipping.

The foam volume was recorded 1 h after whipping to determine foam stability as a percent of initial foam volume.

(8) $$\text{FS}\;\left(\%\right)=\frac{\text{Volume of foam after}\;1\;h}{\text{Volume of foam AW}}\times 100$$

#### 2.5.9 Color

The color of the flour samples was measured with a colorimeter (*3nh Spectrophotometer, NR110, China*), recording coordinates *L* ^*^, *a* ^*^, and *b* ^*^ values. The *L* ^*^ stands for lightness, the *a* ^*^ indicates redness, and the *b* ^*^ represents yellowness (Baidoo et al., 2014). High scores for *L* ^*^, *a* ^*^, and *b* ^*^ correspond to the high degree of lightness, redness, and yellowness, respectively. The total color difference (Δ*E*) was calculated concerning the raw sample from the color parameters *L* ^*^, *a* ^*^, and *b* ^*^ as described in the following equation:

(9) $$\Delta E={\left[{\left(L_{o}‐L\right)}^{2}+{\left(a_{o}‐a\right)}^{2}+{\left(b_{o}‐b\right)}^{2}\right]}^{1/2}$$

where *L*_o_, *a*_o,_ and *b*_o_ are *L* ^*^, *a* ^*^, *b* ^*^ values for the control (raw) sample.

### 2.6 Total polyphenols, total flavonoid, and total antioxidant capacity

#### 2.6.1 Extraction of samples

Extraction was carried out by shaker (*HY‐2(A); Speed Adjusting Multi‐purpose Vibrator, China*) as optimized and reported (Sultana et al., 2009) with slight modification. Ten grams of anchote flour sample was mixed with 100 ml of aqueous methanol solvent (99.8%). The mixture was then shaken for 24 h at room temperature (21°C) and the solution filtered using Whatman Grade 4 filter paper. The final extract stored in a refrigerator (−4°C) until used for analysis.

#### 2.6.2 Total polyphenols content

The total polyphenols content (TPC) of anchote flour extracts was estimated according to the Folin–Ciocalteu method as adopted from Tanvir et al. (2017). Accordingly, 0.4 ml of the extract was mixed with 1.6 ml of 7.5% NaCO_3_ solution, followed by the addition of 2 ml of 10‐fold diluted Folin–Ciocalteu reagent reaction mixture was incubated for one hour in the dark. The intensity of the blue‐colored complex was measured spectrophotometrically at 765 nm wavelength using a spectrophotometer (*V‐630; JASCO, Japan*). Finally, the TPC present in the samples was determined using gallic acid (GAE) standard (*SPECTRUM CHEMICAL, China*) (*R* ^2 ^= .999) as gallic acid equivalent (GAE) in concentration ranges of 12.50–100.00 mg/L and expressed as milligram of GAE/g of the flour sample.

#### 2.6.3 Total flavonoid content

The Total flavonoid content (TFC) of anchote flour extracts was estimated using the colorimetric method adopted from Tanvir et al. (2017). Briefly, 1 ml of extract with concentration of 0.1 g/ml was mixed with 0.3 ml of 5% sodium nitrite. After 5 min, 0.3 ml of 10% aluminum chloride was added, with 2 ml of 1 M sodium hydroxide after 6 min of incubation and with the immediate addition of 2.4 ml of distilled water to produce a total volume of 10 ml. The color intensity of the flavonoid–aluminum complex was measured at 510 nm using a spectrophotometer (*V‐630; JASCO, Japan*). Finally, the TFC was determined against (+)‐catechin standard ( *Sigma‐Aldrich*) (R^2^=0.988) as (+)‐catechin equivalent (CE) in a concentration range of 1.00–100.00 μg/ml, and the result was expressed as milligram of CE/g of anchote flour.

#### 2.6.4 Total antioxidant capacity

The total antioxidant activity of anchote flour was estimated using DPPH (*2, 2′–diphenyl‐1‐picrylhydrazyl*) radical. The radical scavenging activity of the sample extracts was measured using DPPH, according to Woldegiorgis et al. (2014). About 0.004% of DPPH radical solution in methanol was prepared, and then, 4 ml of this solution was mixed with 1 ml of various concentrations (2–16 mg/ml) of the extracts in methanol. The samples were then incubated for 30 min in dark at room temperature (21°C). The scavenging capacity was determined using a spectrophotometer (*JASCO, Japan*) by monitoring the decrease in absorbance at 517 nm. l‐ascorbic acid was used as positive control with the same concentrations as above. The inhibition of radical DPPH in percent (%) was then calculated by using the following equation:

(10) $$\%\;\text{DPPH Inhibition}=\left(\frac{A_{0}‐A_{1}}{A_{0}}\right)\times 100$$

where *A*_0_ is the absorbance of the control reaction and *A*_1_ is the absorbance in the presence of all of the extract samples and reference standards.

### 2.7 Data analysis

The data were subjected to ANOVA, and the significance of the difference between means was determined with Tukey's method at a 95% confidence level (*p* ≤ .05) using Minitab (*Version 16, USA*). The results were expressed as means ± standard deviation of the triplicate treatment. Pearson's correlation coefficient was used to measure the relationship between the flour's properties and the strength and direction of the relationship.

## 3 RESULTS AND DISCUSSION

### 3.1 Anchote flour starch composition

Amylose and amylopectin are the two starch components, and their proportion determines the starch functionality. Amylose and amylopectin contents of anchote starch obtained from its raw and pretreated tuber flours are presented in Table 1. Amylose content of the raw, blanched, and boiled anchote flour starches was ranged in 26.44%–36.11%, 14.18%–21.36%, and 15.52%–16.63%, respectively. In agreement with this, amylose content of 25.5% (raw) and 22.9% (boiled) in plantain flour was reported (Bassey & Dosunmu, 2003). Predrying treatments showed a significant (*p* < .05) decrease in amylose content from raw to blanched and then boiled samples. For instance, at the drying temperature of 60°C, the flour sample from the boiled tuber exhibited a 37% reduction in amylose content than flour from the raw sample dried at the same temperature. Predrying treatments also showed a significant effect on amylopectin content. However, the decrease in amylose was accompanied by an increase in percentage of amylopectin content. Similar findings also showed a reduction in amylose content by boiling in cocoyam (*Xanthosoma maffa (Scoth)*) (raw 25.51%, boiled 23.54%) and aerial yam (*Dioscorea Bulbifera*) (raw 30%, boiled 26.5% ) tubers (Adegunwa et al., 2011; Sanful et al., 2017). The decrease could be due to the disruption of the ordered structure of starch granules leading to the breakdown of the amylose‐amylopectin chain and leaching out of amylose into the water during blanching and boiling (Bassey & Dosunmu, 2003; Guillén et al., 2018; Sanful et al., 2017). The significant decrease in amylose content from the boiled sample compared with the blanched one might be the relatively higher boiling temperature and extended boiling time of the tuber.

**TABLE 1:** Amylose and amylopectin contents of anchote flour starch pretreated and dried at different drying temperatures

PT	DT (°C)	Amylose (%)	Amylopectin (%)
Raw	60	26.4 ± 1.57^c^	73.6 ± 1.57^d^
	80	36.1 ± 0.32^a^	63.9 ± 0.32^f^
	100	31.4 ± 0.47^b^	68.6 ± 0.47^e^
Blanched	60	21.3 ± 0.49^d^	78.6 ± 0.49^c^
	80	19.9 ± 0.04^de^	80.1 ± 0.04^bc^
	100	14.1 ± 2.08^f^	85.8 ± 0.73^a^
Boiled	60	16.6 ± 0.73^ef^	83.4 ± 0.73^ab^
	80	15.5 ± 1.46^f^	84.5 ± 1.46^a^
	100	15.6 ± 3.10^f^	84.4 ± 3.10^a^
LSD (0.05)		2.54	2.54
CV%		6.7	1.9

Note

Values are mean ± standard deviation of three replications. Means shared the same letters in a column are not significantly different (*p* < .05).

Abbreviations: CV, coefficient of variation; DT, drying temperature; LSD, least significant difference; PT, pretreatment.

The drying temperature of pretreated samples also showed a significant difference (*p* < .05), mainly for the raw and blanched samples (Table 1). The highest amylose content (36.1%) was observed for raw samples dried at 80°C and followed by the same sample but dried at 100°C (31.4%) and the lowest (26.4%) for the raw sample dried at 60°C. The relative increase in the percentage of amylose content might be associated with the release of amylose from the amylopectin network due to the destruction of starch granules at higher drying temperatures (Vithu et al., 2020). Blanched samples dried at 60 and 80°C (Table 1) showed no significant difference in amylose and amylopectin contents than samples dried at 100°C. This trend might result from the offset of the released amylose due to the temperature effect by its leaching out during blanching and boiling. However, drying temperature showed no effect on amylose and amylopectin contents for boiled samples. This could be due to the formation of insoluble starch through dissociation and reaggregation of the amylose chain during boiling, which might be less affected by drying temperature (Chen et al., 2017). This implies that drying at the lowest temperature is recommended from an energy‐cost perspective.

The amylose content of the raw anchote starch (26.44%–36.11%) was comparable with previous reports for cassava (23.79%–48.79%), yam (29.5%–30.0%), and water yam (23.35%–33.28%) (Adegunwa et al., 2011; Agyepong & Barimah, 2018; Sanful et al., 2017). The result was higher than the report of Babu and Parimalavalli (2014) for sweet potato starch (18.17%–18.56%). However, greater amylose contents were reported in potato (40.8%), water yam (44.18%), and *Dioscorea pyrifolia* (44.47%) (Arueya & Ojesanmi, 2019; Sharlina et al., 2017; Zhao et al., 2018). The amylose content of anchote starch was in the range normally expected (15%–30%) in starches except the raw tuber dried at higher temperatures (80°C and 100°C), in which it was slightly higher, and for the blanched tuber dried at 100°C, it was lower. The starch amylose content determines its functional properties such as water absorption capacity (WAC), swelling power (SP), thickening, and gelling (Arueya & Ojesanmi, 2019; Babu & Parimalavalli, 2014), which may dictate most of the starch uses. Therefore, the higher amylose content in raw flour starch contributes to its lower functionality, which could limit its application in food processing industries such as bakery.

The higher amylose content of anchote starch could imply a higher degree of crystallinity and thermostability (Egharevba, 2019). It was reported that starches with high amylose contents tend to be of the B‐type crystal structure (Adegunwa et al., 2011). The previously reported B‐type structural pattern of anchote starch is additional confirmation for the higher amylose content (Abera et al., 2019). Anchote starch with high amylase content could be used for firmer, stiffer, and less sticky products (Guillén et al., 2018). Therefore, it can also be used in health products because amylose has been shown to reduce insulin and glycemic response, risk of obesity, cardiovascular disease, and type II diabetes (Sharlina et al., 2017). The raw anchote flour with high amylose could be suitable for use as an additive that increases the crispiness of cracker products. It may also be used in coating and additive industries, which require low permeability to water and good oxygen‐barrier properties (Sharlina et al., 2017).

In contrast, the lower amylose content of the pretreated anchote starch implies its application in industries to produce thickeners and binders. Starch with high amylose exhibits low digestibility because of its less susceptibility to enzymatic hydrolysis (Adegunwa et al., 2011; Agyepong & Barimah, 2018; Egharevba, 2019; Guillén et al., 2018; Sanful et al., 2017). The branched amylopectin exhibited a lower rate of retrogradation but with increased susceptibility to amylase activity (Egharevba, 2019). Since heat‐moisture treatment decreases the starch long chains (Magallanes‐Cruz et al., 2017), digestibility of the pretreated anchote starch could be affected because cooking might contribute to the modification of the starch property.

The pretreated anchote flours with higher amylopectin could be used in food products that might need gelatinization of starches (Alcázar‐Alay & Meireles, 2015; Tortoe et al., 2017a). The pretreated flours such as starch with high amylopectin can also be used to produce a low‐density product since amylopectin‐rich starch greatly expands in size (Yazid et al., 2018). This is confirmed by the relatively higher swelling power of the pretreated anchote flours (Table 2). The result suggested that the pretreated anchote flour with higher amylopectin content might be suited for the production of bakery products: jams and jellies.

**TABLE 2:** Functional properties of raw, blanched, and boiled anchote flour dried at different temperatures

PT	DT (°C)	pH	TSS (°Brix)	WAC (g/g)	OAC (g/g)	WAI (g/g)	WSI (%)	SP (g/g)	FC (%)	FS (%)
Raw	60	6.10 ± 0.01^b^	7.2 ± 0.3^c^	2.72 ± 0.03^f^	1.21 ± 0.04^b^	3.93 + 0.13^e^	13.23 ± 0.75^bcd^	4.99 ± 0.47^d^	33.33 ± 3.05^a^	20.00 ± 0.89^a^
	80	5.98 ± 0.01^cd^	5.8 ± 0.2^d^	2.68 ± 0.07^f^	1.44 ± 0.15^a^	3.91 ± 0.06^e^	13.75 ± 0.29^bc^	4.56 ± 0.49^f^	19.61 ± 1.96^b^	4.92 ± 0.08^b^
	100	5.81 ± 0.01^e^	5.4 ± 0.2^d^	2.42 ± 0.03^g^	1.52 ± 0.02^a^	3.40 ± 0.13^f^	11.40 ± 0.90^d^	5.24 ± 0.03^d^	7.29 ± 0.62^c^	2.47 ± 1.07^b^
Blanched	60	5.97 ± 0.02^cd^	10.8 ± 0.2^a^	3.63 ± 0.08^b^	1.19 ± 0.00^bc^	4.94 ± 0.13^b^	15.17 ± 0.76^b^	6.07 ± 0.07^c^	4.67 ± 1.15^c^	1.91 ± 0.02^b^
	80	5.82 ± 0.01^e^	7.2 ± 0.2^c^	3.35 ± 0.02^d^	1.17 ± 0.02^bc^	4.40 + 0.16^cd^	14.83 ± 1.04^b^	6.14 ± 0.05^c^	4.31 ± 0.60^c^	2.22 ± 0.55^b^
	100	6.02 ± 0.01^c^	6.8 ± 0.2^c^	3.15 ± 0.09^e^	1.07 ± 0.08^c^	4.14 ± 0.07^de^	13.92 ± 0.38^bc^	6.65 ± 0.34^b^	6.00 ± 2.00^c^	1.89 ± 0.04^b^
Boiled	60	6.46 ± 0.01^a^	9.2 ± 0.6^b^	4.21 ± 0.06^a^	1.09 ± 0.03^c^	5.42 ± 0.04^a^	20.37 ± 1.48^a^	6.81 ± 0.24^b^	7.33 ± 1.15^c^	2.48 ± 1.06^b^
	80	6.02 ± 0.01^c^	6.8 ± 0.2^c^	3.72 ± 0.09^b^	1.12 ± 0.08^bc^	4.65 ± 0.35^c^	13.33 ± 0.58^bcd^	7.20 ± 0.03^a^	6.00 ± 2.00^c^	1.89 ± 0.04^b^
	100	5.91 ± 0.01^d^	6.8 ± 0.3^c^	3.54 ± 0.03^bc^	0.94 ± 0.13^d^	4.38 ± 0.05^cd^	12.05 ± 0.43^cd^	6.61 ± 0.27^b^	3.31 ± 1.13^c^	2.55 ± 1.06^b^
LSD (0.05)		0.07	0.74	0.10	0.10	0.42	2.20	0.36	5.17	4.16
CV		1.22	3.74	1.85	6.01	5.42	8.10	4.67	15.62	15.02

Note

Values are mean ± standard deviation of three replications. Means shared the same letters in a column are not significantly different (*p* < .05).

Abbreviations: CV, coefficient of variation; DT, drying temperature; FC, foaming capacity; FS, foam stability; LSD, least significant difference; OAC, oil absorption capacity; PP, preprocessing;SP, swelling power; WAC, water absorption capacity; WAI, water absorption index; WSI, water solubility index.

### 3.2 Functional properties

#### 3.2.1 pH

The pH of flours obtained from raw, blanched, and boiled anchote samples dried at different temperatures ranged from 5.81 to 6.47 (Table 2). The effect of blanching and boiling on the flours' pH values was significant (*p* < .05). On the contrary, the flour's pH was significantly (*p* < .05) decreased with increasing drying temperature, except for the blanched sample. This study illustrated that the flours under this study were slightly acidic (*pH* < 7) compared with that of sweet potato flour (5.8–6.2) (Tortoe et al., 2017b), but slightly higher compared with the pH of yam (5.88), cocoyam (5.22), and plantain (5.74) flours (Oladeji et al., 2013). The pH level dramatically affects flour's performance in many food processing applications. Low pH (<4) flour is not recommended for industrial products, particularly for processing bakery and pastry products (Tortoe et al., 2017b). According to this study, the boiled anchote flour dried at a lower temperature (60°C) with a relatively higher pH (6.46) could be better used as an ingredient in the food industry to formulate food products.

#### 3.2.2 Total soluble solids

The TSS of raw and pretreated anchote flours dried at different temperatures are presented in Table 2. TSS reflect the amounts of water‐soluble solids present in a sample such as sucrose, fructose, vitamins, minerals, amino acids, proteins, organic acids, hormones, and inorganic salts (Borela, 2018). Both pretreatment and drying temperature significantly (*p* < .05) affected the TSS. Blanching and boiling increased the TSS with the highest observed values of 10.8 and 9.2 °Brix, respectively. This finding is in agreement with the report of Iheagwara et al. (2020) for raw and boiled sweet potato flours. The increase compared to flour from the raw tubers might be due to the breakdown of starch into sugars and other soluble molecules during the heat treatments (Thriveni et al., 2019).

On the contrary, an increase in drying temperature reduced TSS values. The reduction could be due to the oxidative breakdown of complex molecules (organic acids, starch, and sugars) into simple molecules such as carbon dioxide and water at higher drying temperatures (Rahman et al., 2019). The absence of significant changes in the TSS value at drying temperatures of 80 and 100°C is consistent with Seifu et al. (2018) report. Compared with other tuber crops, the result of this study is relatively lower than that of TSS of sweet potato varieties (Caetano et al., 2018). However, it is comparable with the TSS reported for fresh sweet potato (5.8) (Prathiksha & Naik, 2017). As compared to flour from raw tuber, the blanched and boiled anchote flours dried at 60°C with relatively high TSS could be used as potential food ingredients to modify the texture of foods since TSS could affect the thickness and texture of food products.

#### 3.2.3 Water absorption capacity

The WAC of anchote tuber flour as affected by predrying treatment and drying temperature is presented in Table 2. Both pretreatment and drying temperature significantly affected (*p* < .05) the WAC of the flours. With an increase in heat treatment during predrying treatment, the WAC increased (boiled > blanched > raw); however, with an increase in drying temperature, the WAC decreased (60 > 80 > 100°C). WAC values in this study are in close agreement with the report of Shebabaw (2013) for raw (2.19 g/g) and boiled (2.51 g/g) anchote flours. A similar trend was also reported in raw (3.00 g/g) and boiled (3.26 g/g) *Dioscorea bulbifera* flours (Aathira & Siddhuraju, 2017) and for blanched yam (Akubor, 2017). The increase could be associated with the heat on the water‐binding sites of anchote proteins, which could produce subunits with more water‐binding sites (Akubor, 2017). This is also in line with the increase in amylopectin content, which binds more water (Table 1). On the contrary, the WAC of the test samples was decreased by increasing drying temperature. This decrease agrees with previous reports for aerial yam, white yam, water yam, and cocoyam flours (Achy et al., 2017; Adegunwa et al., 2011). The decrease might be due to the damage of starch granules and the denaturation of the samples’ protein at higher drying temperatures because of the disruption of hydrogen bonding and nonpolar hydrophobic interactions responsible for WAC (Osobie et al., 2013).

WAC, the ability of a product to incorporate water, is a crucial processing parameter having implications for viscosity, bulking, and consistency of products (Eke‐ejiofor, 2015; Ojo et al., 2017). The higher WAC indicates better flour from functional properties point of view. The WAC of anchote flour in this study was more remarkable compared with that of cassava (1.00) and sweet potato (2.00) flours (Eke‐ejiofor, 2015). However, it was comparable with *Dioscorea rotundata*, *Dioscorea alata,* and *Dioscorea bulbifera* flours (3.10–3.90) (Amandikwa et al., 2015). The relatively higher WAC of anchote flour may be due to the high polar amino acid residues of protein and carbohydrates having an affinity for water molecules (Ojo et al., 2017). The increased WAC in food systems enables end‐users to manipulate the dough's functional properties in bakery products (Eke‐ejiofor, 2015). High WAC could also be advantageous in baby food formulation, whereas lower WAC is desirable for making gruels (Ojo et al., 2017). The determined WAC of anchote flours suggested that the boiled flour was better used to formulate baby foods and bakery products than the raw.

#### 3.2.4 Oil absorption capacity

Oil absorption capacity is an important parameter as it improves the mouthfeel and retains food products' flavor (Haruna et al., 2019). It is dependent on the surface availability of hydrophobic fats and amino acids (Kazeem et al., 2018). The OAC of anchote flours obtained from raw and pretreated tubers dried at different temperatures is presented in Table 2. According to the results, both pretreatment and drying temperature significantly affected (*p* < .05) OAC. Blanching and boiling reduced OAC as compared to the raw sample, which agrees with the results reported for *Dioscorea bulbifera* (raw 4.52, boiled 4.11 g/g) and cocoyam (raw 2.03, blanched 1.99 g/g) flours (Aathira & Siddhuraju, 2017; Akinlua et al., 2018). The decrease in OAC could be due to the degradation and decline of hydrophobic amino acids responsible for oil binding.

On the contrary, the effect of drying temperature on OAC is less significant for blanched and boiled tuber samples. OAC was increased with an increase in drying temperature for the raw anchote flour. The increase might be due to a critical loss of moisture during drying at higher temperatures (Haruna et al., 2019), thereby allowing more oil to be absorbed. In this study, a decrease in the WAC of the same sample observed with increasing temperature agrees with this concept since WAC and OAC are contradicting in the properties.

When the OAC of anchote flour in this study (0.94–1.52 g/g) is compared with other tuber crops, it is comparable with the report of Akubor (2017) for raw (0.97) and blanched (1.08) yam flour, but it is less than that of orange‐fleshed sweet potato (1.7–2.1 g/g) flour (Akinlua et al., 2018). However, values in the present finding are greater than the OAC of potato (0.63 g/g) flour (Navale et al., 2016). According to this study, the raw anchote flour dried at a higher temperature could retain its natural flavor better than the blanched and boiled flours.

#### 3.2.5 Water absorption index (WAI)

The WAI of raw, blanched, and boiled anchote flours dried at varying temperatures is presented in Table 2. The result showed that pretreatments (blanching and boiling) and drying temperature significantly affected (*p* < .05) the WAI of the flours. The WAI of the boiled anchote flour is the highest (60°C), and that of the raw sample is the lowest. The higher WAI of boiled anchote tuber indicates the flour's tendency to be gelatinized and well‐cooked (Osibanjo et al., 2017). A similar increase in WAI after blanching was also reported for three orange‐fleshed sweet potato tuber flours (Osibanjo et al., 2017).

On the contrary, the WAI of the flours decreased with increasing drying temperature. Our result is in agreement with Ahmed et al. (2009) for sweet potato flour. The increase might be due to a relative increase in protein and carbohydrate contents, level of starch damage, pore size, capillary, and protein charges (Jamal et al., 2016; Omoniyi et al., 2016). It is because hydration involves the interaction of hydrophilic components of the flour by hydrogen bonding with water molecules (Jamal et al., 2016).

Higher WAI values point out the weak association of starch polymers in the native granule requiring much water during reconstitution (Osibanjo et al., 2017). The WAI observed in pretreated anchote flours (3.40–5.42 g/g) is higher than in sweet potato (2.35 g/g) flour (Omoniyi et al., 2016) but comparable with that of wheat (4.76 g/g) and rice (5.38–6.26 g/g) flours (Jamal et al., 2016; Kumar & Saini, 2016).

#### 3.2.6 Water solubility index

Water solubility index of the flours was significantly (*p* < .05) affected by predrying treatments and drying temperature, the highest value for boiled anchote flour and the lowest for the raw (Table 2). The values increased after blanching and boiling treatments (strong effect) but decreased with an increase in the drying temperature (less effect) (Table 2). The higher the solubility, the higher the protein's functionalities in food (Ige, 2017). The highest WSI was observed in the blanched and boiled anchote tubers dried at a lower temperature (60°C). This result is in line with results reported in orange‐fleshed sweet potato tuber flours (Osibanjo et al., 2017), and the increases observed in aerial yam (*Dioscorea bulbifera*) flour from 17.31% to 36.85% after boiling (Achy et al., 2017). In agreement with the trend in our result, a progressive decrease in solubility of orange‐fleshed sweet potato tuber flour was also reported within the drying temperature range of 40–60°C (Haruna et al., 2019). The decrease in solubility with an increase in drying temperature could be due to critical loss of moisture and progression from fine flour into ashing level at higher temperature (Haruna et al., 2019).

However, the WSI of anchote flour in this study is higher than in cassava (1.24%), plantain (3.88%), maize (0.70%), and baking wheat (4.67%) flours (Arisa et al., 2017; Ige, 2017; Onyango et al., 2013). The higher WSI can be advantageous in masking or reducing undesirable taste effects from other components in a given product (Osibanjo et al., 2017). Flours with high water solubility resulting in low paste viscosities should be targeted at weaning and specialty foods (Olatunde et al., 2016). Therefore, anchote flour with the relatively higher WSI, mainly obtained from blanched and boiled tuber dried at a lower temperature, could be used for such food products.

#### 3.2.7 Swelling power

Swelling power measures the hydration capacity because food eating quality is often associated with the water retention in the swollen starch granules (Vengaiah et al., 2013). The average SP of the raw, blanched, and boiled anchote flours dried at various temperatures ranged between 4.56 and 7.20 g/g (Table 2). Blanching and boiling had significantly (*p* <.05) increased the SP of the resulting flours. Similar increases were reported for the SP of raw (6.75 g/g) and blanched (8.04 g/g) cocoyam flour (Akinlua et al., 2018). The enhanced SP of the flours could be due to an increase in hydrophilic groups as a result of heat treatment during blanching and boiling (Ukom et al., 2018). On the contrary, the effect of drying temperature on the SP of the flours was not statistically significant (*p* >.05). The result revealed that higher SP was recorded in the boiled flour and lower in the raw. The SP of anchote flour fall in the range reported for sweet potato varieties (4.5–7.9 g/g) (Lai et al., 2016). However, it is higher compared with sweet potato (1.42 g/g) and lower relative to that of cassava (9.76 g/g) flours (Omoniyi et al., 2016; Onyango et al., 2013).

The SP of flour might be affected by amylose and protein contents, which might inhibit the granular swelling due to disulfide and intermolecular bonding in protein (Jamal et al., 2016). This agrees with Table 1, where high amylose content in raw flour corresponds to low SP. This property may influence bakery product characteristics, as flours having lower SP may cause the bakery product not to swell well (Kusumayanti et al., 2015). The SP of anchote flour is lower than that of the commonly used baking ingredient wheat flour (11.01 g/g) (Alviola & Monterde, 2018). As a result, anchote may need modification or mixing with flours having high SP to be used as an ingredient for bakery products.

#### 3.2.8 Foaming capacity and foam stability

The foaming capacity and foaming stability of anchote flour for both raw and pretreated samples are presented in Table 2. The result showed that FC of the raw flour was significantly (*p* < .05) different from both blanched and boiled flours. The highest FC was observed in the raw anchote flour dried at 60°C (33.33%), whereas the lowest was in boiled flour dried at 100°C (3.31%). This variation implies that FC of the flour is critically affected by heat treatments during predrying treatment and by drying temperature. Results in this study are also in agreement with the results of Harijono et al. (2013) for raw (44.67% ) and blanched (16.67%) purple water yam flours. Similarly, Akubor (2017) reported FC of 14% (raw) and 2.0% (blanched) in yam flours. The negative effect of drying temperature to decrease FC of bread fruit flour from 800% (60°C) to 200% (70°C) was also reported by Kazeem et al. (2018). The decrease in FC might result from thermal degradation of protein due to blanching/boiling and elevated drying temperatures, since the higher FC is attributed to high protein content due to proteins' surface‐active properties to entrap gas bubbles (Harijono et al., 2013; Ukom et al., 2018).

The findings showed that the highest FS was observed in the raw anchote flour dried at 60°C (20.0%), while the lowest FS was observed in boiled flour dried at 80°C (Table 2). The result illustrated that pretreatment significantly (*p* < .05) affected FS at 60°C, while drying temperature significantly (*p* < .05) affected FS of the raw tuber flour. Increasing drying temperature reduced the FS of the raw tuber flours. Harijono et al. (2013) reported a reduction in protein content by blanching, and hence, FS decreased in yam flours due to denaturation of proteins that may lead to its coagulation. FS is related to protein content because some proteins have surface‐active properties to entrap gas bubbles. Their water solubility determines the ability of proteins to stabilize foaming since soluble protein can reduce surface tension at the interface between air bubbles and surrounded liquid (Harijono et al., 2013). Therefore, FS might be influenced by the level of solubilized protein and the content of polar and nonpolar lipids in a sample. Generally, the result revealed that a better FC and FS were obtained in raw anchote flour dried at a lower temperature (60°C). A product with excellent FC and FS values can be utilized as a substitute in the production of foam‐forming foods. The raw anchote flour can be used for such purposes as compared to the blanched and boiled.

#### 3.2.9 Color

The lightness (*L* ^*^), redness (*a* ^*^), yellowness (*b* ^*^), and total color change (Δ*E*) values for the raw and pretreated anchote flours are presented in Table 3. The *L* ^*^ values of the raw anchote flour were significantly (*p* < .05) lower than in the pretreated flours, which implies that blanching/boiling positively impacted the flour's brightness. Similarly, an increase in drying temperature significantly (*p* < .05) increased the brightness of the flour. Better lightness (*L* ^*^ values) for pretreated samples as compared to the raw might be due to the less effect of enzymatic browning, which could be caused by phenolase enzyme (Ngoma et al., 2019). The finding is in agreement with the report of Nascimento and Canteri (2018), in which blanching enhanced the *L* ^*^ value of potato flour from 46.72 to 55.26. The values of a color coordinate *a* ^*^ were also affected by pretreatment temperature but not significantly (*p* > .05) affected by drying temperature. The decrease in redness after blanching/boiling is in line with what was reported for potato flour (Nascimento & Canteri, 2018). An increase in drying temperature increased the *b* ^*^ value in the raw anchote flour but reduced in the blanched and boiled samples. High yellowness intensity was observed in the flour obtained from blanched and boiled tuber dried at 60°C. In agreement with this, an increase in *b* ^*^ value by blanching was reported for water yam (*Dioscorea alata*) flour (Harijono et al., 2013). The result showed that the color of blanched and boiled flours was lighter than in the raw.

**TABLE 3:** Effect of pretreatment and drying temperature on color parameters of anchote flour

PT	DT (°C)	*L* ^*^	*a* ^*^	*b* ^*^	Δ*E*
Raw	60	51.96 ± 1.39^d^	4.28 ± 0.72^ab^	11.49 ± 0.81^c^	0
	80	65.73 ± 0.54^c^	5.03 ± 0.46^a^	12.84 ± 0.31^bc^	0
	100	74.82 ± 1.59^b^	4.23 ± 0.47^ab^	14.02 ± 0.75^b^	0
Blanched	60	78.04 ± 1.33^ab^	4.02 ± 0.37^ab^	16.55 ± 0.30^a^	26.59 ± 2.49^a^
	80	78.92 ± 1.86^a^	3.57 ± 0.65^abc^	13.38 ± 0.35^b^	13.29 ± 2.30^b^
	100	78.32 ± 0.54^ab^	3.53 ± 0.76^abc^	13.67 ± 0.69^b^	3.62 ± 1.57^c^
Boiled	60	79.81 ± 1.43^a^	4.00 ± 0.71^ab^	16.14 ± 0.82^a^	28.26 ± 1.25^a^
	80	81.15 ± 0.88^a^	2.24 ± 0.07^c^	13.29 ± 0.53^b^	15.69 ± 0.56^b^
	100	79.81 ± 1.3^a^	2.79 ± 0.27^bc^	12.94 ± 0.22^bc^	5.44 ± 2.10^c^
LSD (0.05)		2.20	0.93	1.75	9.79
CV%		1.70	9.50	7.67	6.67

Note

Values are mean ±standard deviation of three replications. Means shared the same letters in a column are not significantly different (*p* < .05). *L* ^* ^= degree of brightness; *a* ^* ^= degree of redness; *b* ^*^ = degree of yellowness; Δ*E* = total color difference.

Abbreviations: CV, coefficient of variation; DT, drying temperature; LSD, least significant difference; PT, pretreatment.

Color changes mainly occur due to pigment loss and enzymatic and nonenzymatic reactions during drying (Liu et al., 2019). Compared with the raw, the mean total color change (Δ*E*) in anchote flour due to blanching and boiling of the tuber ranged from 3.62 to 28.26 (Table 3). The result revealed that Δ*E* significantly differed (*p* < .05), suggesting a difference in the color of the control and the pretreatments of anchote flour. This observation agrees with what was reported for blanched taro flour (Baidoo et al., 2014). However, no significant color difference was found between the blanched and boiled flours. The Δ*E* was significantly (*p* < .05) decreased with increasing drying temperature. This coincides with that reported in lily bulb flours in which ΔE linearly decreased with increasing drying temperature (Liu et al., 2019). Generally, the lowest color change was found in the blanched flour dried at 100°C relative to its corresponding raw flour.

### 3.3 Total polyphenols, flavonoid, and total antioxidant capacity

#### 3.3.1 Total polyphenol content

**FIGURE 2:**
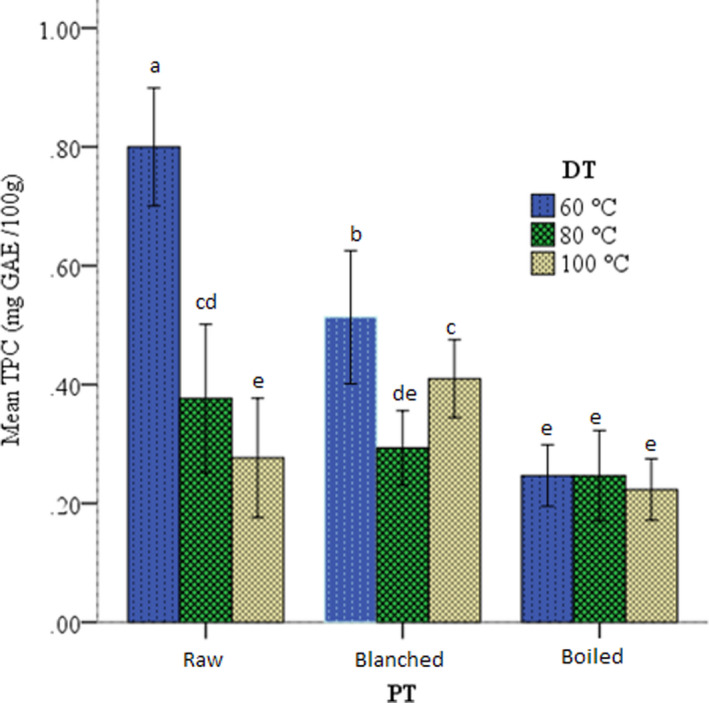
Effect of predrying treatments (PT) on TPC of anchote flour dried at different drying temperatures (DT)

Polyphenols are common constituents of plant‐origin food products, and they have functional and health advantages. The Total polyphenol content (TPC) level of raw and pretreated anchote flours is presented in Figure 2. Both predrying treatment and drying temperature showed a significant impact on TPC of the flours. The highest TPC (0.80 mg GAE/100 g) was observed in the raw anchote flour dried at a relatively lower temperature (60°C), whereas the lowest TPC (0.22 mg GAE/100 g) was observed in the boiled anchote flour dried at 100°C. This result implies that the higher heat treatment during pretreatment and drying results in the lower TPC. Similar results were reported for Cocoyam (*Xanthosoma maffa (Scoth)*) flour in which TPC of 0.78, 0.46, and 0.29 mg/100 g for the raw, blanched, and boiled products, respectively (Ukom et al., 2018). Kuyu et al. (2018) also reported similar trends in sweet potatoes. Polyphenol loss increased with an increase in drying temperature, which might be associated with the thermal degradation of the polyphenols.

The TPC of anchote flour in this study (0.22–0.80 mg GAE/g) is lower than in the previous report for anchote accessions (Ayalew, 2016). The difference could be ecology, variety, soil fertility, and maturity that significantly affect the TPC (Hamouz et al., 2006; Omar et al., 2012). However, the result lies in the range of TPC for yam and cocoyam (0.07–1.63 mg GAE/g) as reported by Ukom et al. (2014). On the contrary, the higher TPC was recorded in *Dioscorea alata* (6.8 mg/g), and even greater TPC was reported in sweet potato (10.5–119 mg GAE/g) (Fidrianny et al., 2018; Sakthidevi & Mohan, 2013). Anchote flour contains lower TP than other tuber crops, and the raw anchote flour dried at lower temperature preserved more TPC.

#### 3.3.2 Total flavonoid content

**FIGURE 3:**
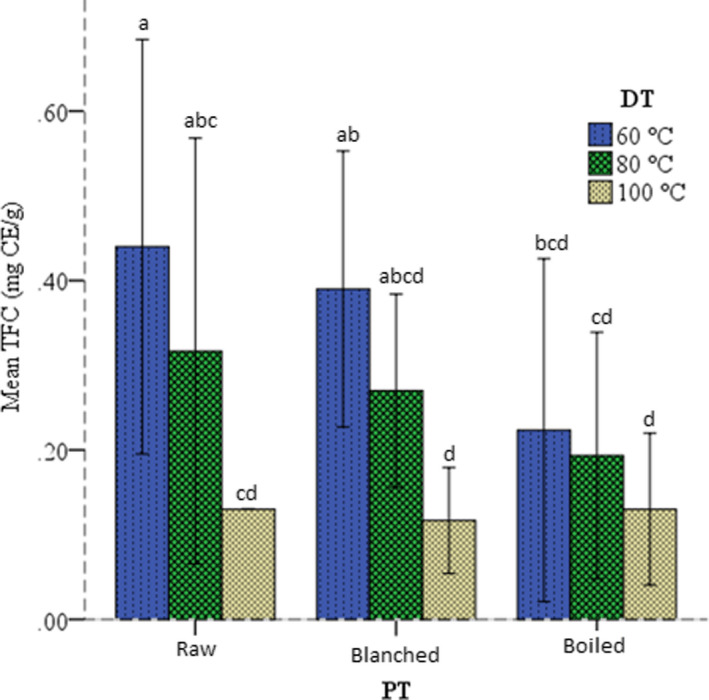
Effect of pretreatment (PT) on TFC of anchote flour dried at different drying temperatures (DT)

Flavonoids are significant components of phenolic compounds with high human health benefits because they have more potent antioxidant activity than other phenolic groups due to multiple hydroxyl groups in their structure (D'Amelia et al., 2018). The TFC of raw, blanched, and boiled anchote flours dried at different temperatures is presented in Figure 3. The values of TFC in the anchote flour samples ranged between 0.12 and 0.44 mg CE/g. Higher TFC was observed in raw anchote dried at 60°C and lower in the blanched sample dried at 100°C (Figure 3). This implies that both pretreatment and drying temperature significantly (*p* < .05) affected the TFC of the flours. The TFC was reduced by blanching/boiling and with increasing drying temperature. This finding agrees with the report of Ukom et al. (2018), in which the flavonoid content of flours prepared from raw, blanched, and boiled cocoyam was 0.4, 0.2, and 0.14 mg/100 g, respectively. The decrease may be due to thermal decomposition of the compounds during the heat treatments.

The TFC in the present finding is similar to the range reported by Ayalew (2016) for anchote accessions (0.41–0.63 mg CE/g). The slight variation may be due to production ecology, variety, and soil fertility (Hamouz et al., 2006; Omar et al., 2012). The result also lies in the range of 0.031–1.55 mg CE/g reported for yam and cocoyam (Ukom et al., 2014). On the contrary, it is lower than that obtained for *Dioscorea alata* (12.1 mg/g) and sweet potato (6–178 mg QE/g) tubers’ extracts (Fidrianny et al., 2018; Sakthidevi & Mohan, 2013). This comparison might show that anchote flour contains relatively low TF, though they have immense dietary importance as phytonutrients. From this finding, it is possible to conclude that minimizing the impacts of pretreatment could contribute to retaining more flavonoids in anchote flour.

#### 3.3.3 Total antioxidant capacity

**FIGURE 4:**
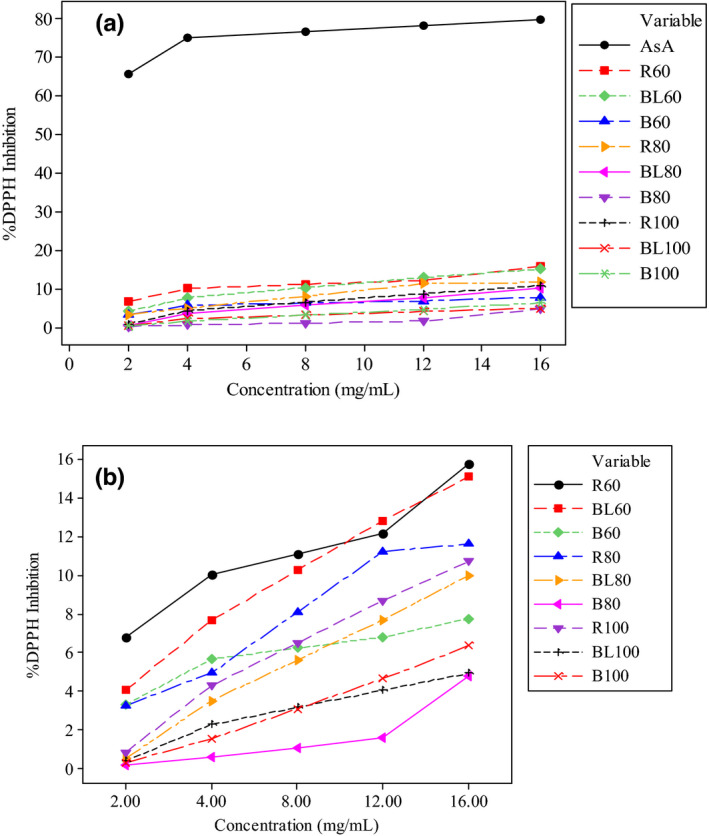
Comparison of anchote flours’ DPPH scavenging activity with ascorbic acid standard (a) and effect of pretreatment on the flours’ DPPH scavenging activity (b) (AsA—ascorbic acid, R_60_—raw dried at 60°C, BL_60_—blanched dried at 60°C, B_60_—boiled dried at 60°C, R_80_—raw dried at 80°C, BL_80_—blanched dried at 80°C, B_80_—boiled dried at 80°C, R_100_—raw dried at 100°C, BL_100_—blanched dried at 100°C, B_100_—boiled dried at 100°C)

Antioxidants are substances that inhibit oxidation by reaction with free radicals and the decomposition of free radicals forming lipid hydroperoxides (Curayag et al., 2019). The antioxidant activities of anchote flour extracts as affected by predrying treatment and drying temperature were evaluated by determining the ability to scavenge DPPH‐free radicals. The capacity of the flours to scavenge the free radical was compared with the activity of a standard ascorbic acid (Figure 4). The result revealed that all the tuber flour extracts had a deficient radical scavenging activity relative to the standard, which is expected from low‐moisture dried products compared with fresh fruits and vegetables. Figure 4 shows that the antioxidant activity of the flours was decreased by pretreatments and increasing drying temperature. This observation agrees with the decrease in antioxidant activity of purple yam flour by processing (Larief et al., 2018) and orange‐fleshed sweet potato by increasing drying temperature (Kuyu et al., 2018). The decrease may be due to the heat instability of the antioxidant components of the tuber when treated with hot water and dried at higher temperature (Kuyu et al., 2018; Larief et al., 2018). The result showed that the polyphenol content directly contributes to the antioxidant activity potential of the flours. The antioxidant activity of raw anchote flour dried at a lower temperature (60°C) was comparatively higher due to more polyphenolic compounds.

### 3.4 Correlation between selected flour parameters

The relationship between physical and functional variables measured for anchote tuber flours was evaluated using Pearson's correlation coefficient, as presented in Table 4. Accordingly, WAC showed a strong positive correlation with WAI (*r* = .968) and SP (*r* = .798), while negatively correlated with OAC (*r *= −.794). This relation agrees with the negative correlation reported between the WAC and OAC in rice and maize flours (Wani & Kumar, 2015). A strong correlation between WAC and SP was also reported in potatoes flours (Klang et al., 2019). The OAC of flours, in turn, showed a significant (*p* < .001) negative correlation with WAI (*r* = −.735) and SP (*r* = −.730), where WAI exhibited a strong positive association with SP (*r* = .787). The *L* ^*^ showed a strong negative correlation with TPC (*r* = −.808) of the flours. The FC and FS of the flours were highly correlated (*r* = .908), and both of them showed a positive correlation with TPC and TFC. This might be because of the dependence of TFC on protein content (Ukom et al., 2018), which decreased by pretreatment and increasing drying temperature (Bikila et al., 2020). The correlation study also revealed that AC was negatively correlated with SP (*r* = −.89) and WAC (*r* = −.76) of the anchote flour, which is in agreement with the reports of Babu and Parimalavalli (2014) in sweet potato and Chisenga et al. (2019) in cassava.

**TABLE 4:** Pearson's correlation coefficients among the measured parameters of anchote flour properties

	WAC	OAC	WAI	SP	FC	FS	TPC	L*	AC
WAC	1								
OAC	−0.794^a^	1							
WAI	0.968^a^	−0.736^a^	1						
SP	0.798^a^	−0.730^a^	0.787^a^	1					
FC	−0.503^c^	0.282	−0.539^c^	−0.699^a^	1				
FS	−0.427^c^	0.070	−0.485^c^	−0.488^c^	0.908^a^	1			
TPC	−0.375	0.125	−0.452^c^	−0.480^c^	0.758^a^	0.851^a^	1		
L*	0.624^b^	−0.372	0.477^c^	0.739^a^	−0.964^a^	−0.918^a^	−0.808^a^	1	
AC	−0.76^a^	0.858^a^	−0.615^b^	−0.892^a^	0.559^c^	0.286	0.272	−0.608^b^	1

Note

L* = whiteness.

Abbreviations: AC, amylose content; FC, foaming capacity; FS, foam stability; OAC, oil absorption capacity; SP, swelling power; TPC, total polyphenol content; WAC, water absorption capacity; WAI, water absorption index.

^a^Significant *p* ≤ .0001; ^b^significant *p* ≤ .001; ^c^significant *p* ≤ .05.

The correlation analysis showed that the flour functional properties WAC, WAI, and SP changed in a similar pattern by the treatments. In contrast, the other groups OAC, WSI, FC, FS, and AC were affected negatively by the same factors. The result showed that the treatments (blanching/boiling and drying temperature) increase one of the parameters WAC, WAI, and SP would increase the other and vice versa. These parameters increased by blanching and boiling compared with the raw. Similarly, any change in either WSI, FC, FS, AC, or TPC of the flours due to pretreatment and drying temperature would change the others in a similar trend.

## 4 CONCLUSIONS

Both predrying treatments and drying temperatures exhibited an impact on the amylose and amylopectin ratio. Relatively higher amylose ration was observed in a flour from raw anchote, which can be used to produce firmer, stiffer, and less stick food products. The higher the amylose ratio could also correspond with the presence of more resistant starch for less digestibility of the product for better health benefits associated with noncommunicable chronic diseases. However, the lower amylose from pretreated flours can be used as a thickener and binder in food industries. Predrying treatments also showed a relatively positive impact on TSS, WAC, WAI, WSI, SP, and L* values of the flours. Tuber pretreatments (blanching/boiling) can be recommended in food application if these functional properties are considered for commercial use. In contrast, if the interest is to get better amylose, FC, FS, TPC, TFC, and more total antioxidant capacity, it is better to use flours from raw tuber without pretreatments. In general, an increase in drying temperature resulted in a more negative impact on the measured parameters. The lower the drying temperature (60°C) is, the better the result for measured parameters, regardless of pretreatments.

## CONFLICT OF INTEREST

The authors have no conflict of interest to declare.

## AUTHOR CONTRIBUTIONS

**Adugna Mosissa Bikila:** Conceptualization (equal); Data curation (lead); Formal analysis (equal); Funding acquisition (equal); Investigation (lead); Methodology (equal); Project administration (equal); Software (equal); Validation (equal); Visualization (equal); Writing – original draft (lead); Writing – review & editing (equal). **Yetenayet Bekele Tola:** Conceptualization (equal); Data curation (equal); Formal analysis (equal); Funding acquisition (equal); Investigation (equal); Methodology (equal); Project administration (lead); Resources (equal); Software (equal); Supervision (equal); Validation (equal); Visualization (equal); Writing – review & editing (equal). **Tarekegn Berhanu Esho:** Project administration (supporting); Supervision (supporting); Validation (supporting); Visualization (supporting); Writing – review & editing (equal). **Sirawdink Fikreyesus Forsido:** Conceptualization (equal); Formal analysis (supporting); Funding acquisition (supporting); Investigation (equal); Methodology (equal); Project administration (equal); Resources (equal); Software (equal); Supervision (supporting); Validation (equal); Visualization (equal); Writing – review & editing (equal). **Desta Fekadu Mijena:** Data curation (supporting); Resources (supporting).

## ETHICAL APPROVAL

This study did not involve any human or animal testing.

## Data Availability

The data that support the findings of this study are available from the corresponding author upon reasonable request.
